# SciFi detector and associated method for real‐time determination of profile and output factor for small fields in stereotactic radiotherapy

**DOI:** 10.1002/mp.14019

**Published:** 2020-01-30

**Authors:** P. Pittet, J. Esteves, J.‐M. Galvan, G.‐N. Lu, F. Blanc, G. Haefeli, P. Hopchev, S. Rit, L. Desbat, J. Ribouton, P. Jalade

**Affiliations:** ^1^ Institut des Nanotechnologies de Lyon INL CNRS UMR5270 Université de Lyon Université Claude Bernard Lyon 1 F‐69100 Villeurbanne France; ^2^ Laboratoire de Physique des Hautes Energies LPHE EPFL CH‐1015 Lausanne Switzerland; ^3^ University Lyon INSA‐Lyon Université Claude Bernard Lyon 1 CNRS UMR 5220 Inserm U1206 CREATIS Lyon France; ^4^ University Grenoble Alpes CNRS Grenoble INP TIMC‐IMAG F‐38000 Grenoble France; ^5^ Service de Radiophysique et Radiovigilance Hospices Civils de Lyon Centre Hospitalier Lyon Sud F‐69495 Pierre‐Bénite France

**Keywords:** scintillating detectors, small‐field dosimetry

## Abstract

**Purpose:**

For determining small‐field profile and output factor during stereotactic radiotherapy quality assurance (QA) procedures, we propose a novel system based on the scintillating fiber (SciFi) detector with output image acquisition and processing to allow real‐time monitoring of profile and output factor.

**Materials and methods:**

The employed detector is a SciFi detector made of tissue‐equivalent scintillating plastic fibers arranged in 6‐layer fiber ribbons with a fiber pitch of 275 μm in each layer. The scintillating signal at the detector output is acquired by a sCMOS (scientific complementary metal–oxide–semiconductor) camera and represents the projected field profile along the fibers axis. An iterative reconstruction method of the field from its projected profile based on a priori knowledge of some features of the radiation field defined by the stereotactic cones is suggested. The detector with implemented data processing has been tested in clinical conditions, for determining beam profiles and output factors, using cone collimators of different sizes from 4 to 15 mm diameter. The detector under test was placed at 1.4 cm depth and 98.6 cm source to surface distance (SSD) in a water‐equivalent phantom and irradiated by a 6 MV photon beam.

**Results:**

The reconstructed field profiles obtained from the detector are coherent with data from EBT3 radiochromic films, with differences within ±0.32 mm for both the FWHM and the penumbra region. For real‐time determination of the field output factor, the measured data are also in good agreement with data independently determined by the French Institute for Radiological Protection and Nuclear Safety (IRSN) based on radiochromic films and thermoluminescent 1 × 1 mm^2^ micro‐cubes dosimeters (TLD). The differences are within ±1.6% for all the tested cone sizes.

**Conclusions:**

We propose and have tested a SciFi plastic scintillating detector with an optimized signal processing method to characterize small fields defined by cone collimators. It allows the determination of key field parameters such as full width at half maximum (FWHM) and field output factors. The results are consistent with those independently measured using TLD and radiochromic films. As the SciFi detector does not require a correction factor, it is in line with the International Atomic Energy Agency (IAEA) and the American Association of Physicists in Medicine (AAPM) TRS‐483 recommendations, and can be suitable for online QA of small radiation fields used in photon beam radiotherapy, and is compatible with MRI‐LINAC.

## 1 Introduction

In modern radiation therapy, small static photon fields for the treatment of intra‐ and extracranial tumors have increasingly been used. They are associated with at least one of the following physical conditions: (a) There is a loss of lateral charged particle equilibrium (as the field sizes approach that of the lateral secondary electron range) and (b) there is partial occlusion of the primary photon source by the collimating devices on the beam axis.^1^, ^2^, ^3^

Small fields are obtained by flattened or un‐flattened high‐energy photon beams shaped either with micro‐multileaf collimators (MLCs) or with a set of conical collimators. The use of cones is typically preferable for targets smaller than the leaf width since they provide higher mechanical stability and sharper dose falloff compared to micro‐MLC.^4^

Due to the profound clinical consequences of incorrect beam data, there has been a strong demand for systematic and independent assessment of small radiation fields during commissioning and periodic quality assurance (QA) procedures.^5^ However, such procedures are time‐consuming tasks to implement and therefore remain particularly challenging. They require the use of appropriately small detectors with accurate positioning to limit the volume‐averaging effect on the large dose gradients associated with the small fields.^3^ Moreover, small‐field related partial occlusion of primary photon source, loss of lateral charged particle equilibrium and the perturbation of the charged particle fluence can become a serious issue for non‐tissue‐equivalent detectors.^3^

To address these problems, there has been a very dynamic research activity over the last decade, in particular concerning the determination of the output factor (OF) which is a critical dosimetric parameter for the characterization of small fields used in radiotherapy.^4^, ^6^, ^7^, ^8^, ^9^

Correction factors have been proposed for small‐field dosimetry based on solid‐state detectors such as diodes and microdiamonds.^8^, ^10^, ^11^ However, in the recent joint IAEA — AAPM publication of the International Code of Practice TRS‐483, the use of detectors that exhibit output correction factors close to unity is recommended for the field output factor determination.^3^ Radiochromic films and plastic scintillation detectors fulfill this recommendation and are of particular interest for small‐field dosimetry.^12^, ^13^, ^14^ However, radiochromic films are two‐dimensional (2D) detectors requiring post‐processing and they do not allow real‐time QA procedures. Plastic detection scintillators are mainly implemented as point detectors and thus require (a) an accurate positioning in the field for the output factor determination (which can be delicate to implement) and (b) a 2D displacement of the point detector for scanning the irradiated area, which is not compatible with real‐time requirements.

The aim of the study reported here was to explore the use of the scintillating fiber (SciFi) detector, a 2D highly spatially resolved plastic scintillating detector (initially developed for the LHCb experiment at CERN) with an optimized signal processing method for small‐field commissioning and QA procedures.^15^, ^16^ Our investigation focuses on this detector to measure output factors of small static fields defined by stereotactic cones in 6 MV photon beams. The obtained results are compared with those obtained independently from Institute for Radiological Protection and Nuclear Safety (IRSN, France) with both radiochromic films and micro‐cubes thermoluminescent dosimeters, as recommended for small‐field monitoring by Bassinet et al.^17^

## 2 Materials and Methods

### 2.1 The SciFi scintillating detector and signal acquisition

**Figure 1:**
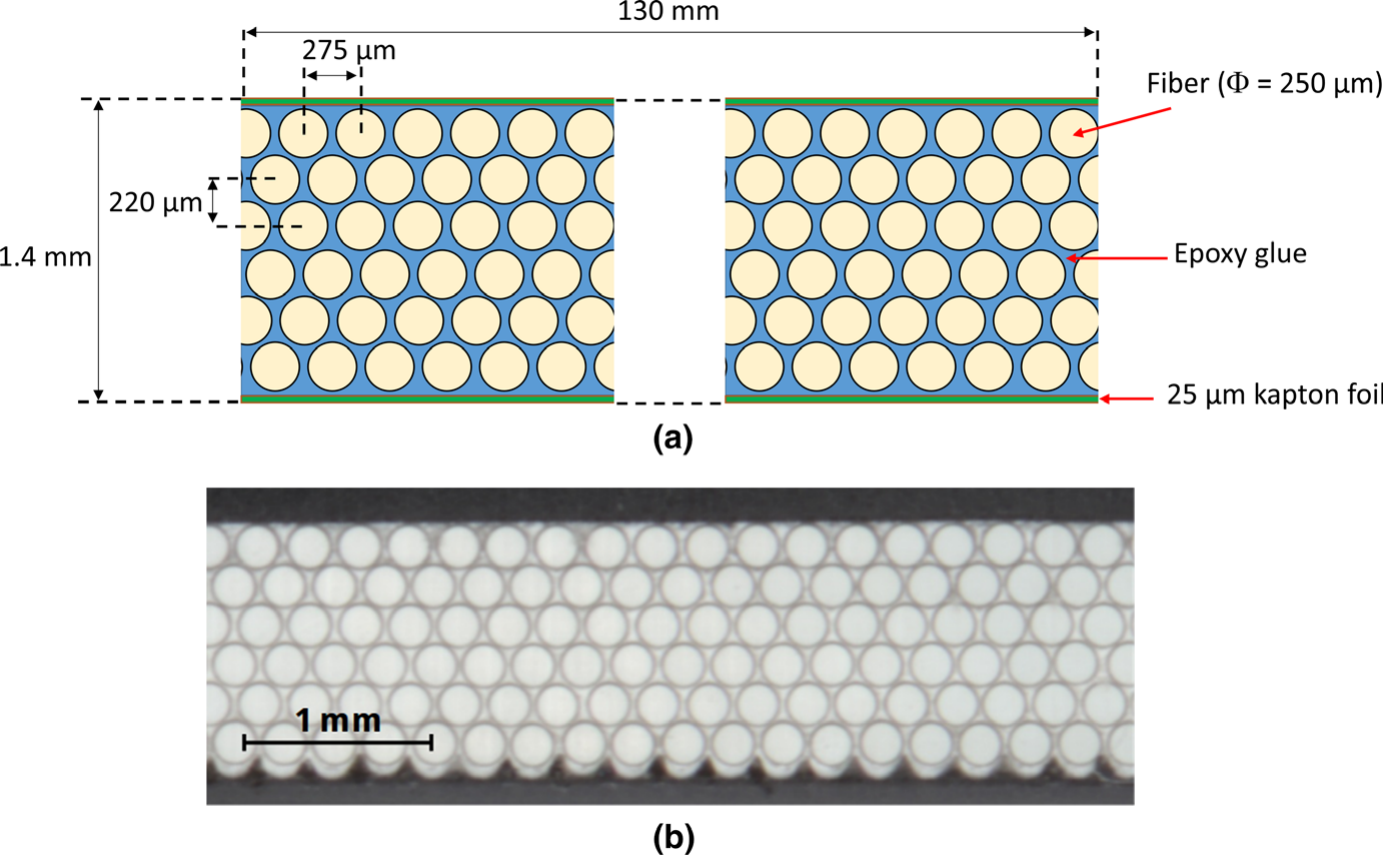
Cross section of the Scifi detector^15^, ^16^: (a) schematic view and (b) photograph. [Color figure can be viewed at http://wileyonlinelibrary.com]

The SciFi detector initially developed for the LHCb experiment at CERN is made of tissue‐equivalent, 250 μm in diameter, polystyrene scintillating fibers (SCSF‐78MJ, Kuraray, Japan) arranged in a staggered close‐packed geometry to 6‐layer fiber ribbons (with a fiber pitch of 275 μm in each layer) as shown in Fig. 1.^15^, ^16^ The radioluminescence signal at the fibers output has a spectrum from 415 to 550 nm, and peaks at 450 nm.^15^ The fibers have been bonded with a titanium dioxide (20% in weight) loaded two‐component epoxy which fills the gap between the fibers and reduces the optical cross talk between the waveguides. The detector is covered by 25 µm thick Kapton foils on both top and bottom faces for required stiffness in practical use. 3M Enhanced Specular Reflector (ESR) film is glued on one end of the ribbon to maximize scintillation signal detection at the other end of the ribbon. The detector manufacturing is described in the *LHCb‐PUB‐2015‐008* CERN report.^16^ The SciFi detector prototype is 40 cm in length, 13 cm in width, and 1.4 mm in thickness.

**Figure 2:**
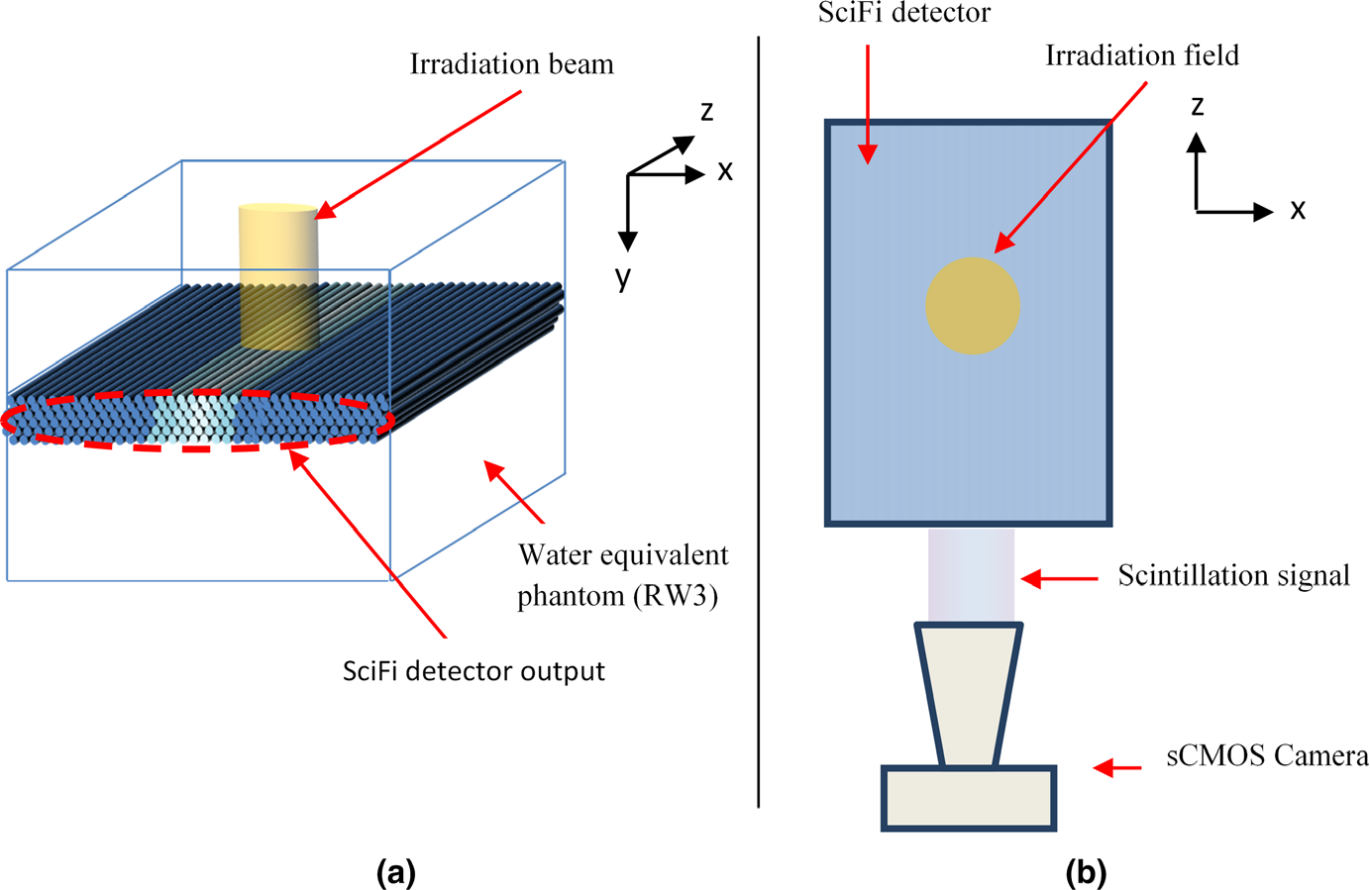
Measurement setup: (a) isometric and (b) top views (DICOM patient coordinate system is also displayed on the images). [Color figure can be viewed at http://wileyonlinelibrary.com]

Figure 2 illustrates small‐field monitoring using SciFi detector and a sCMOS camera (Zyla 5.5, Andor‐Oxford Instruments Ltd, UK) equipped with a macro video lens (Zoom 7000 from Navitar Inc., USA). The SciFi detector is placed in the plane perpendicular to the beam axis, with center alignment. Under radiation, each fiber output of the detector gives an integrated scintillating signal, and the detector output provides a profile of integrated scintillating signals. The camera (with connected laptop) serves to acquire image for signal monitoring, storage, and processing. It is noted that each layer of SciFi detector gives a fiber pitch of 275 µm and that fibers in an adjacent layer are arranged to be shifted by half of a pitch. The acquired 6‐layer image allows a resolution of 137.5 µm in the direction orthogonal to the beam assuming that the deposited dose is the same in each layer.

Since the camera is placed inside of the medical LINAC vault, radiation‐induced transient noise appears on the captured image as sharp spikes or impulses affecting one or a small cluster of pixels, and thus, a 3 × 3 median filter pre‐processing method is applied on the acquired images to reduce the impulse noise contribution.^18^

To further improve SNR (gain of 14 dB), a 5 × 5 image binning is applied at the expense of image resolution. However, the image resolution remains acceptable for the SciFi detector’s resolution (~140 µm).^19^

Considering the DICOM coordinate system (illustrated in Fig. 2), the profile of scintillating signals at the output of SciFi detector, $p\left(x\right)$, is determined on image by y‐axis integration. This profile corresponds to a one‐dimensional (1D) projection of the radiation field in z‐axis direction.

The obtained data are used as input for the proposed reconstruction algorithm that will be described later.

### 2.2 Experiment conditions

All measurements presented in this paper have been carried out at the radiotherapy department of the University Hospital of Lyon (Centre Hospitalier Lyon Sud). Radiation is provided from Novalis TRUBEAM STX (Varian Medical Systems Inc, USA) to deliver 6 MV flattened photon beam at a dose rate of 600 MU/min. A set of stereotactic conical collimators with nominal diameters of 15, 12.5, 10, 7.5, 5 and 4 mm are employed. When stereotactic cones are attached to the LINAC, the field size defined by the jaws is set to 3 × 3 cm^2^ in service mode and kept constant for all the cone sizes.

The SciFi detector is placed in a solid water phantom at a source‐to‐detector distance (SSD) of 100 cm and depth of maximum dose (1.4 cm). These experimental conditions have been used by IRSN, and thus, we have adopted it for comparison of our results with EBT3/TLD data from IRSN. The phantom size is 30 × 30 cm^2^ to provide the proper scattering conditions, and 10‐cm slabs are used for backscatter. The SciFi detector and the phantom are positioned to be aligned with the room lasers conventionally employed to project the LINAC axis and isocenter for patient positioning. This ensures the radiation field to be within the detector area.

For measurements using 3 × 3 cm^2^ square field, the SciFi detector can be irradiated with different orientations by changing the collimator angle.

Each captured image is obtained by integrating scintillating signal at the SciFi detector output over 2 s, which corresponds to 20 MU at the LINAC output. This integration time has been chosen, on the one hand, to ensure sufficient signal magnitude and, on the other, to limit the number of pixels affected by radiation‐induced transient noise as mentioned above.

The acquired data are compared with those independently obtained by IRSN using the same equipment following a protocol based on passive dosimeters.^14^ Their protocol implemented radiochromic films (EBT3 Gafchromic®, Ashland Advanced Materials Inc., USA) and micro‐cubes (1 mm × 1 mm × 1 mm) LiF:Mg,Ti thermoluminescent dosimeters (TLD‐700, Harshaw, USA). Four films and four TLD dosimeters were used for each field size, and the output factors were determined over two measurement campaigns. This protocol for film dosimetry aimed at minimizing uncertainties of data especially from mishandling of films.^14^

### 2.3 Approach of SciFi detector data processing for dose profile reconstruction

It is worth mentioning that the radioluminescence signal at the output of each fiber of the SciFi detector results from signal integration along the irradiated fiber length weighted by its optical attenuation and scintillation efficiency.^20^ Thus, each acquired image from the SciFi detector output corresponds to the dose distribution profile of the irradiation field projected along the SciFi detector axis. It is possible to acquire several images with different orientations of the detector in the field to allow tomographic dose reconstruction as initially proposed by Goulet et al.^20^ However, for a small field shaped with any stereotactic cone, we can use the a priori knowledge of the symmetry of revolution of the field for the implementation of a direct field reconstruction from a single image of the SciFi detector output. This approach is simpler to implement and does not suffer from SciFi detector‐repositioning uncertainties nor from eventual dose rate variations during detector repositioning.

It is noted that the projected dose profile from the acquired image is centered. If this profile remains the same for any orientation in the field, it will be possible to use Algebraic Reconstruction Techniques (ART) for instance with the Simultaneous Iterative Reconstruction Technique (SIRT algorithm) or total variation minimization iterative reconstruction algorithm.^20^, ^21^ However, such an approach applied to our case is quite sensitive to noise. For improvements, we propose a more robust algorithm which takes into account two following aspects: (a) revolution symmetry of the reconstruction field; (b) dose decrease with the off‐axis distance.

**Figure 3:**
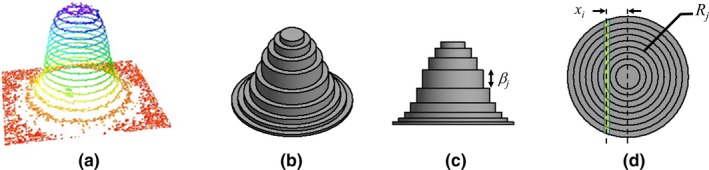
(a) Three‐dimensional surface plot (isolines) of EBT3 film image for the field obtained with the 6‐mm cone and proposed model for the dose distribution obtained with stereotactic cones (b) perspective (c) side and (d) top schematic views. One fiber of the SciFi detector is shown in yellow on this schematic at off‐axis distance of $x_{i}$. [Color figure can be viewed at http://wileyonlinelibrary.com]

In our approach, we assume that the dose distribution at the detector depth in the field and in the penumbra can be approximated by a superimposition of disks of decreasing diameters whose thickness represents a fraction of the dose as illustrated in Fig. 3. The center of these disks is determined by the center of gravity of the projected dose profiles and corresponds to the beam axis.

Based on this assumption, the reconstruction algorithm is simplified to mainly determination of the thickness of each disk.

Thus, considering m disks of strictly increasing radius ${\left.R_{j}\right|}_{j=1..m}$ with $R_{1}>0$, the integrated dose over the ith waveguide of the SciFi detector can be written as a function of disk thickness ${\left.\beta_{j}\right|}_{j=1..m}$:

(1) $$S_{i}=2\sum_{j=1}^{m}\delta_{\mathrm{ij}}\beta_{j}\sqrt{R_{j}^{2}-x_{i}^{2}}$$

where $\left\{\begin{array}{cc} \delta_{\mathrm{ij}}=1 & \mathrm{if}\;x_{i}\leq R_{j}\\ \delta_{\mathrm{ij}}=0 & \mathrm{otherwise} \end{array}\right.$ and $x_{i}$ is the distance of the ith waveguide from the beam axis with $0\leq x_{1}\leq x_{2}\leq \ldots \leq x_{n-1}\leq x_{n}$.

It is noted that if the SciFi detector is not limited to one half of the field but covers the full radiation field, there is a second waveguide at a distance $x_{i}$ from the beam axis if i≠1. In that case, $S_{i}$ corresponds to the average of the signal measured at the two waveguide outputs.

The SciFi detector output can be expressed in matrix form as follows:

(2) $$\left[\begin{array}{c} S_{1}\\ S_{2}\\ \vdots \\ S_{n-1}\\ S_{n} \end{array}\right]=\left[\begin{array}{ccccc} a_{11} & 0 & & 0 & 0\\ a_{22} & a_{22} & \cdots & 0 & 0\\ \vdots & \vdots & \ddots & \vdots & \vdots \\ a_{n-11} & a_{n-12} & \cdots & a_{n-1\;m-1} & 0\\ a_{n1} & a_{n2} & & a_{n\;m-1} & a_{\mathrm{nm}} \end{array}\right]\left[\begin{array}{c} \beta_{1}\\ \beta_{2}\\ \vdots \\ \beta_{m-1}\\ \beta_{m} \end{array}\right]$$

with

(3) $$a_{\mathrm{ij}}=2\delta_{\mathrm{ij}}\sqrt{R_{j}^{2}-x_{i}^{2}}$$

We adopt an iterative resolution of this overdetermined linear system Aβ=S(n≥m), to minimize the norm ||Aβ-S|| with a non‐negative constraint on $\beta_{j}$. We solve it using Simultaneous Iterative Reconstruction Technique (SIRT).^22^ However, this is an ill‐conditioned inverse problem which requires the use of a regularization method to obtain a smooth and stable solution in the presence of noisy data. The adopted regularization method consists in limiting the number of iterations in the SIRT algorithm.^23^

The update equations are as follows:

(4) $$\beta_{k+1}=\left[\begin{array}{c} \begin{array}{c} \beta_{1,k+1}\\ \beta_{2,k+1} \end{array}\\ \vdots \\ \begin{array}{c} \beta_{m-1,k+1}\\ \beta_{m,k+1} \end{array} \end{array}\right]=\beta_{k}+{\mathrm{CA}}^{T}R\left(S-A\beta_{k}\right)$$

and

(5) $${\left.\text{If}\;\beta_{j,k+1}<0,\;\text{then}\;\beta_{j,k+1}=0\right|}_{j=1..m}$$

where C and R are the diagonal matrix that contain the inverse of the sum of the columns and rows of the system matrix A, respectively, that is, $c_{\mathrm{jj}}=1/\sum_{i}a_{\mathrm{ij}}$ and $r_{\mathrm{ii}}=1/\sum_{j}a_{\mathrm{ij}}$.

To reconstruct the small fields (defined by cone collimators) from the one‐dimensional projected profiles, we implement the SIRT algorithm with the following parameters for computations:

- n=123 which corresponds to the 24.6 mm wide projected profile by considering the 200 µm sampling of the profile
- $R_{j+1}=R_{j}+200\;\mu m$, with $R_{0}=400\;\mu m$ and $R_{122}=24.8\;\text{mm}$ (i.e., m=122 which allows a full field coverage)
- Nb_it = 7000 iterations

The initial condition is $\beta_{0}=\left[\begin{array}{c} \begin{array}{c} \beta_{1,0}\\ \beta_{2,0} \end{array}\\ \vdots \\ \begin{array}{c} \beta_{m-1,0}\\ \beta_{m,0} \end{array} \end{array}\right]=\left[\begin{array}{c} \begin{array}{c} 0\\ 0 \end{array}\\ \vdots \\ \begin{array}{c} 0\\ 0 \end{array} \end{array}\right].$

The reconstructed dose profile, $p_{r}\left(x\right)$, is computed by using the following relationship:

(6) $$p_{r}\left(x\right)=\sum_{j=1}^{m}\delta_{x,j}\beta_{j,Nb\_it}\sqrt{R_{j}^{2}-x^{2}}$$

where $\left\{\begin{array}{cc} \delta_{\mathrm{xj}}=1 & if\;x\leq R_{j}\\ \delta_{\mathrm{xj}}=0 & \mathrm{otherwhise} \end{array}\right.$

A reconstructed image of the field can also be computed by using the symmetry of revolution of the field. Each pixel’s gray level, $I_{r}\left(x,z\right)$ in the DICOM coordinate system can be calculated as follows:

(7) $$I_{r}\left(x,z\right)=p_{r}\left(\sqrt{x^{2}+z^{2}}\right)$$

The computation time for iterative reconstruction of the field is much shorter than 1 s using a core i5 laptop (Intel Core i5‐8350U processor), which is acceptable for real‐time field monitoring.

## 3 Results

### 3.1 Signal at SciFi detector output for 3 × 3 cm^2^ field size

**Figure 4:**
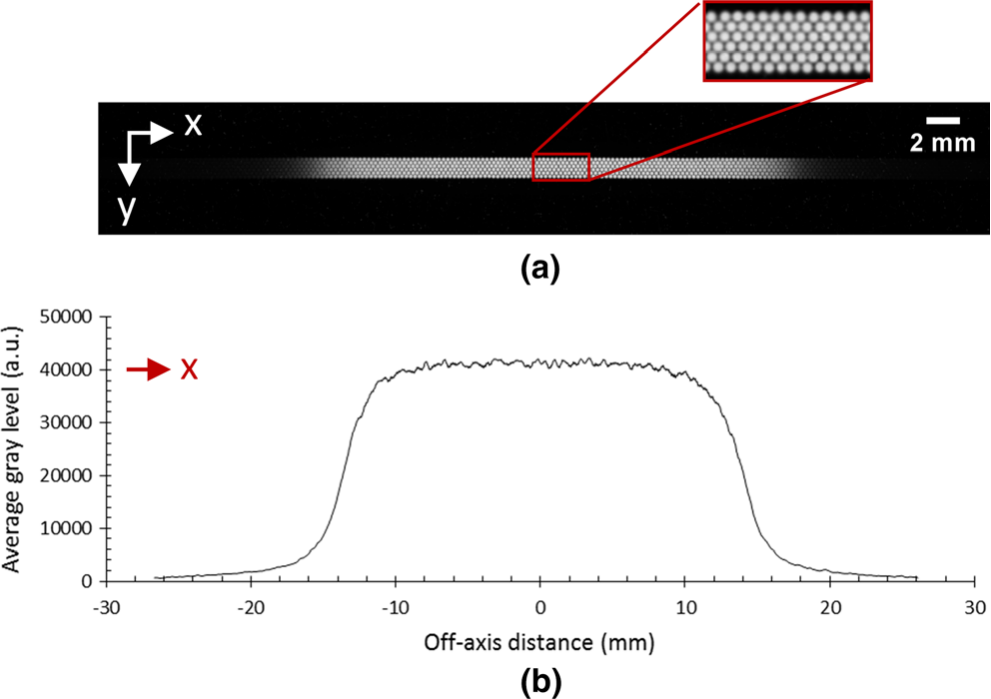
(a) Signal at the output of the scintillating fiber prototype irradiated by a 3 × 3 cm^2^ field, with a zoom showing the fibers arrangement in the detector and (b) the one‐dimensional projected profile, $p\left(x\right)$, determined on image by y‐axis integration (DICOM patient coordinate system is also displayed on the images). [Color figure can be viewed at http://wileyonlinelibrary.com]

Figure 4(a) shows a raw image from the SciFi detector irradiated by a 3 × 3 cm^2^ square field. It allows distinction of each irradiated fiber, with a good signal to noise ratio for image processing. Figure 4(b) plots the corresponding 1D projected profile, $p\left(x\right)$.

**Figure 5:**
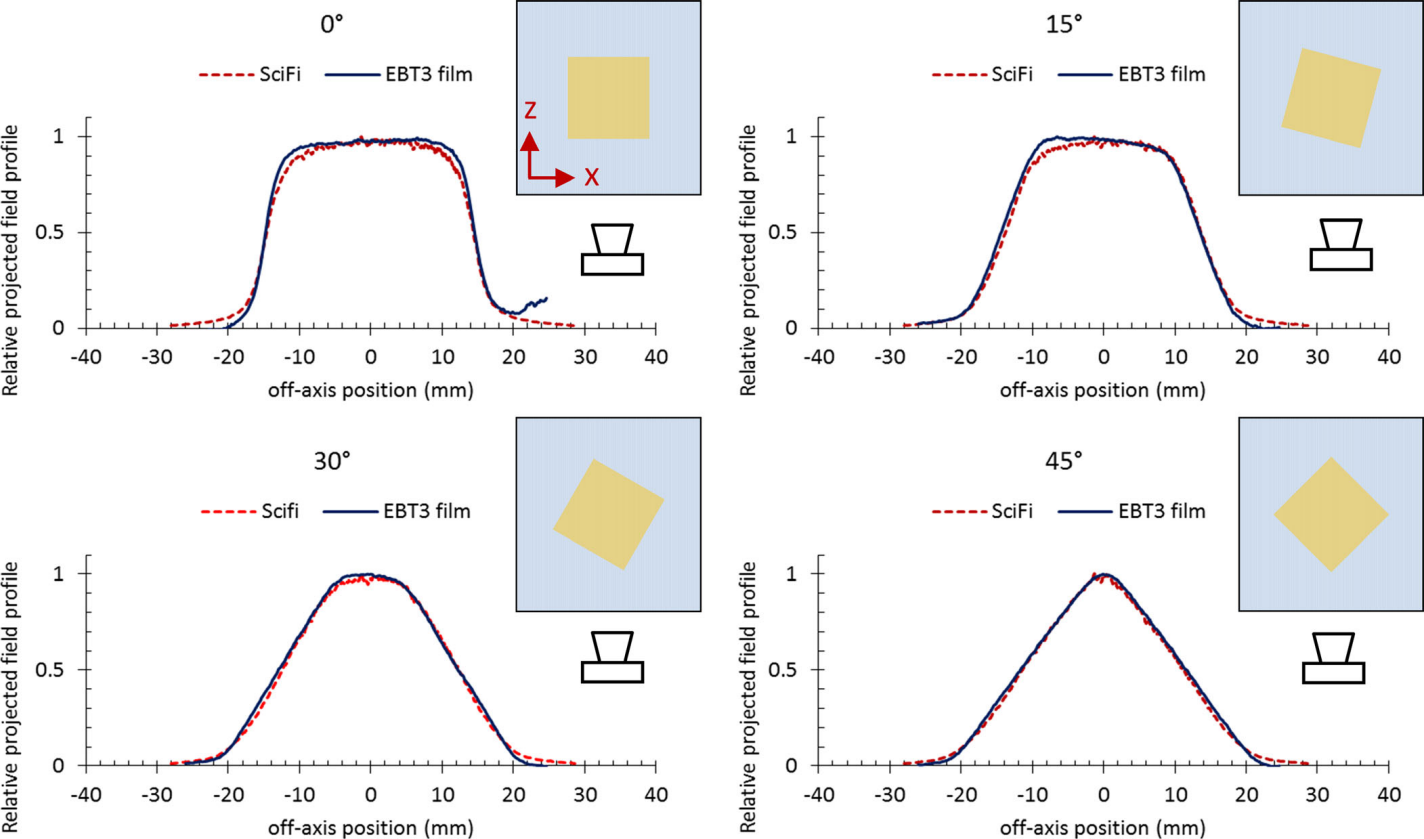
One‐dimensional projected field profiles,$p\left(x\right)$, for different 3 × 3 cm^2^ field orientations, measured using the SciFi detector in comparison with the ones computed based on EBT3 film images (for each plot, the schematic view of the experimental setup. [Color figure can be viewed at http://wileyonlinelibrary.com]

Measurements have been made with the SciFi detector irradiated at various collimator orientation angles from 0° to 45°. Figure 5 shows the 1D projected profile corresponding to each set angle, in comparison with profile determined by using EBT3 film for measurements in the same conditions. There is a good agreement between SciFi and EBT3 data for all field orientations.

### 3.2 Signal at SciFi detector output for stereotactic conical collimators

**Figure 6:**
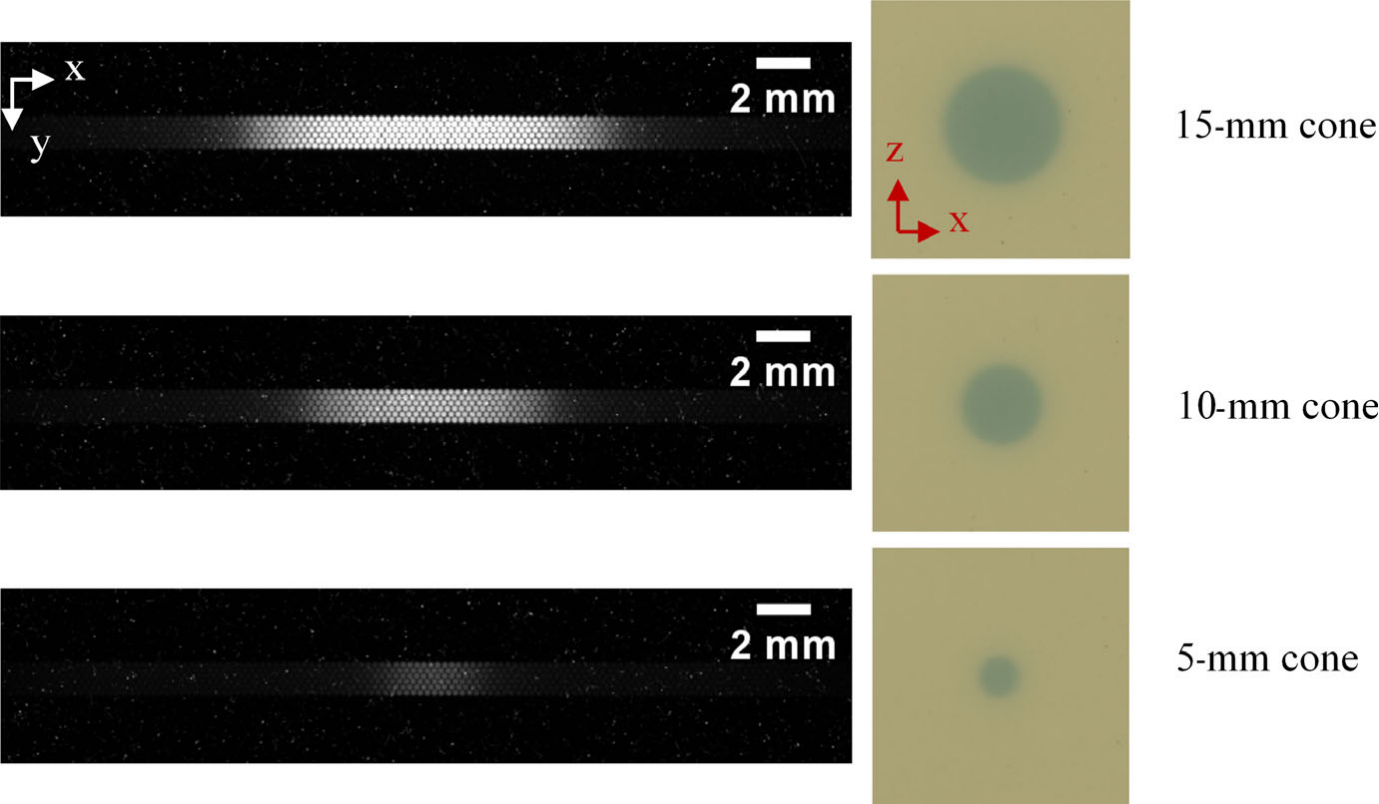
Signal at the scintillating fiber detector output and EBT3 film images for fields obtained with 5‐, 10‐, and 15‐mm cones. [Color figure can be viewed at http://wileyonlinelibrary.com]

The SciFi detector has also been tested in other fields by using 4‐, 5‐, 6‐, 7.5‐, 10‐, 12.5‐, and 15‐mm stereotactic conical collimators. Figure 6 compares signals at the SciFi detector output, and EBT3 film images for fields obtained with 5‐, 10‐, and 15‐mm cones.

**Figure 7:**
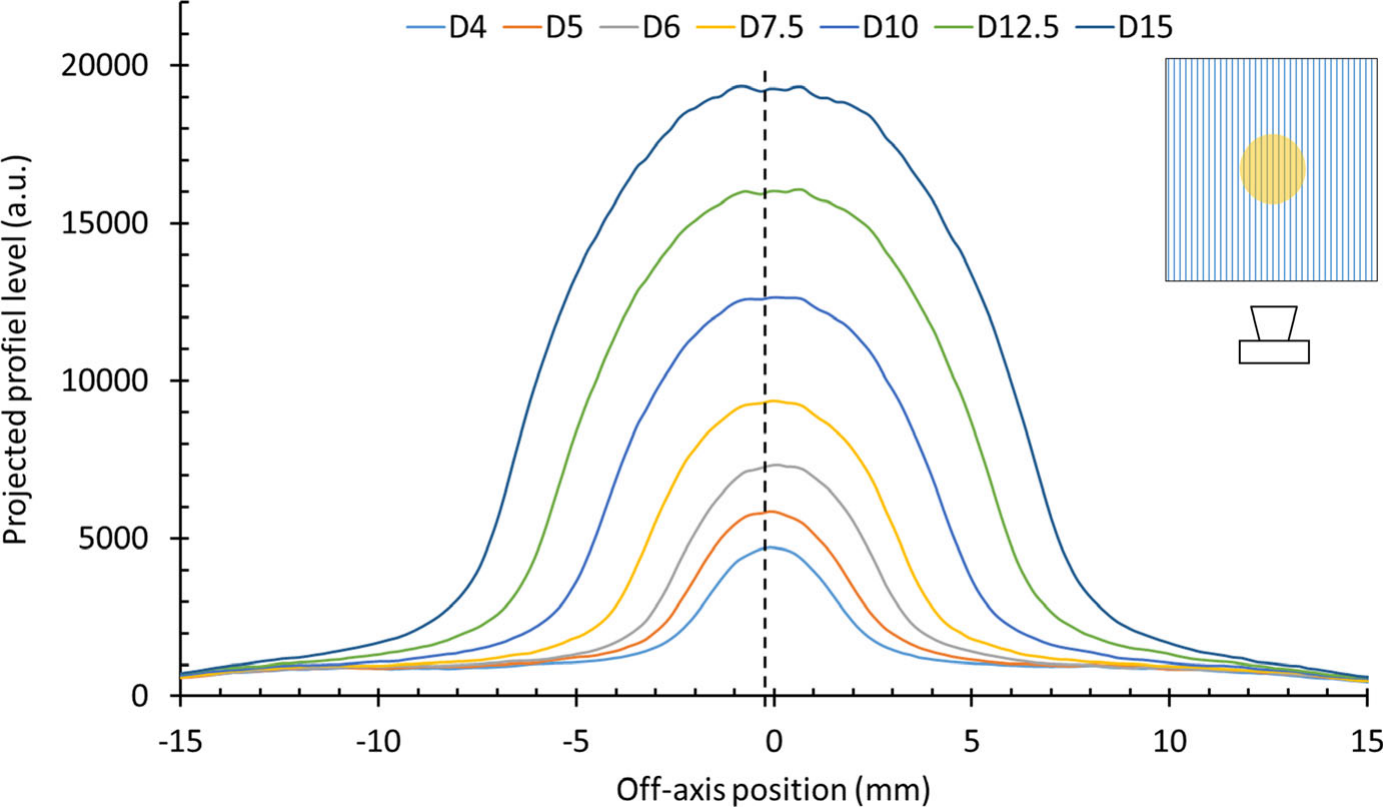
1D projected profiles, $p\left(x\right)$, corresponding to the signal at the SciFi detector output for fields obtained with the 4‐, 5‐, 6‐, 7.5‐, 10‐, 12.5‐, and 15‐mm conical collimators. [Color figure can be viewed at http://wileyonlinelibrary.com]

We can observe that the signal level at the SciFi detector output decreases when the field becomes smaller, as shown in Fig. 7. This is because smaller fields mean shorter lengths of the SciFi scintillating waveguides under radiation, thus measuring weaker signals. However, the signal at the detector’s output remains measurable even for the 4‐mm cone with a signal to noise ratio sufficient for reliable dose profile reconstruction as shown later.

It is worth mentioning that the detector and the phantom have been maintained in the same position for these measurements when changing conic collimators. These results can serve to quantify any displacement of the field center caused by collimator mounting.

To evaluate field center shift due to collimator mounting, we have processed 5 images of the detector output per conical collimator to determine the field center from the 1D projected profiles, $p\left(x\right)$. Table 1 presents statistical results on the field center position. The standard deviation is smaller than 0.02 mm, indicating a good reproducibility of this method to determine the field center. Moreover, the measured reproducibility of conical collimator insertion into mount reported in Table 1 is within the system specifications (Maximum at ±0.2 mm — Expected performance at ±0.1 mm).

**Table 1:** Field center deviations measured with the scintillating fiber detector on 5 images per field.

Cone (mm)	Mean off‐axis position (mm)	Standard deviation (mm)
4	0.11	0.02
5	−0.10	0.01
6	0.05	0.01
7.5	−0.05	0.00
10	−0.01	0.01
12.5	0.01	0.01
15	−0.02	0.01

We have also processed these data according to the method proposed in Section 2.C. It consists in applying a 5 × 5 binning on the SciFi images to increase the signal to noise ratio before the relative dose profile reconstruction processing.

**Figure 8:**
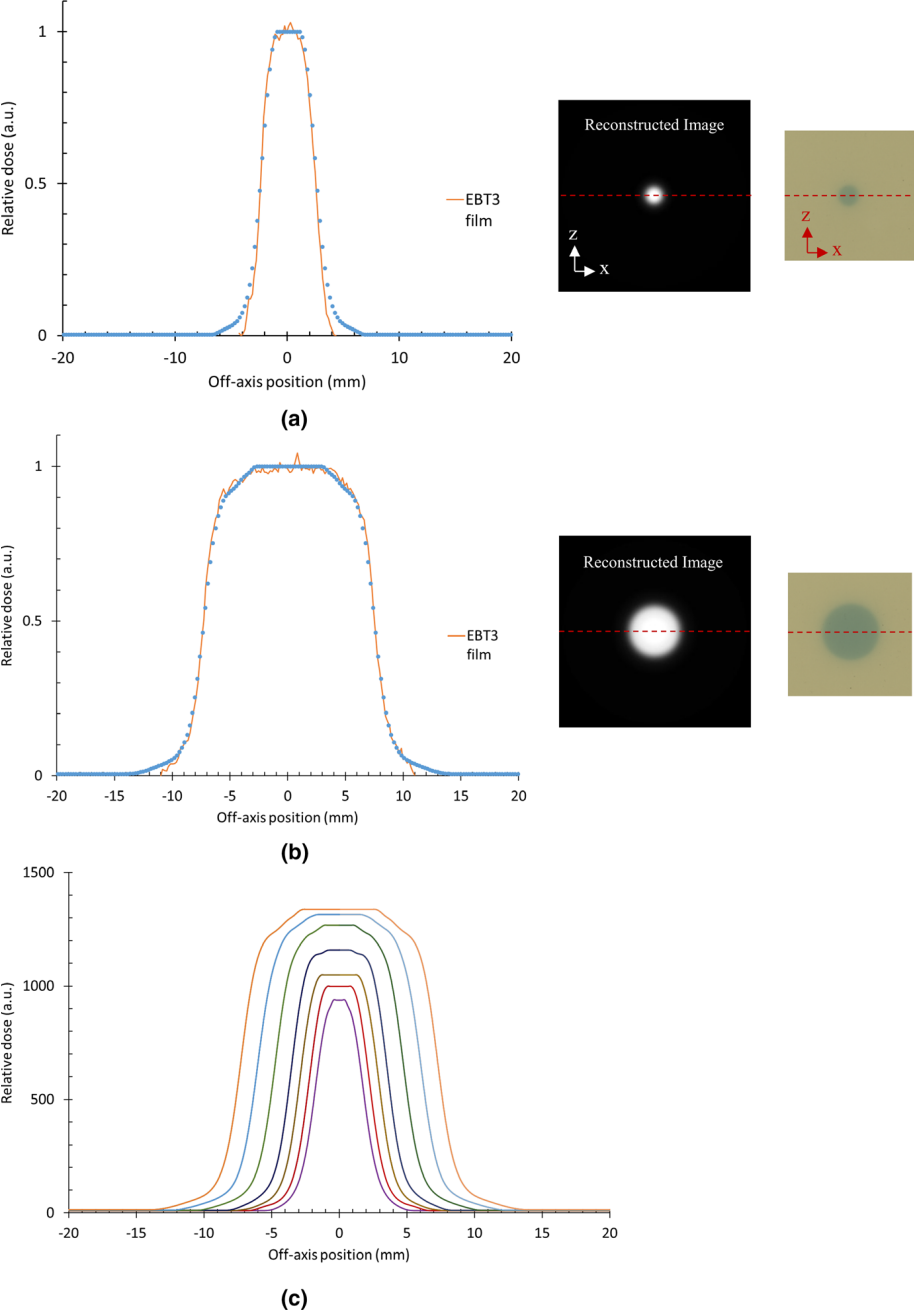
Reconstructed profile, $p_{r}\left(x\right)$ (a) for 5‐mm and (b) 15‐mm circular fields compared with experimental results from EBT3 film (red dashed lines on the film and reconstructed images show the line used for the profile determination). Reconstructed profiles for different field sizes are drawn in Fig. 8(c). [Color figure can be viewed at http://wileyonlinelibrary.com]

For 5‐mm circular field, the reconstructed profile, $p_{r}\left(x\right)$, is compared with experimental results from EBT3 film in Fig. 8(a). For 15‐mm circular field, the comparison is shown in Fig. 8(b) A good agreement is observed for all field sizes. The reconstructed profiles for different field sizes are drawn in Fig. 8(c).

The beam profile parameters (FWHM and penumbra width 20%–80%) can be calculated for each cone size for the SciFi detector and the EBT3 film and are shown in Table 2. It is noted that the 20%–80% penumbra width is calculated as the average between the 20% and 80% ascending and descending parts on the beam profiles as proposed by M. Petasecca et al.^24^ The results obtained with the SciFi detector in comparison with EBT3 measurements exhibit small differences, within +0 µm/−300 µm and +320 µm/−20 µm for FWHM and penumbra, respectively.

**Table 2:** Beam profile parameters [full width at half maximum (FWHM) and penumbra width 20%–80%] determined with (a) the scintillating fiber (SciFi) detector by using the dose profile reconstruction method and (b) EBT3 films.

Cone (mm)		4	5	6	7.5	10	12.5	15
FWHM (mm)	SciFi	4.03	4.92	5.82	7.61	9.85	12.53	14.77
	EBT3	NA	5.08	6.10	7.79	9.99	12.53	15.07
	difference	NA	−0.16	−0.28	−0.18	−0.14	0.00	−0.30
Penumbra (mm)	SciFi	1.57	1.57	1.57	1.79	2.01	2.01	2.24
	EBT3	NA	1.27	1.52	1.52	1.69	2.03	2.03
	difference	NA	0.30	0.04	0.27	0.32	−0.02	0.21

For each circular field, the on‐axis dose can be determined from the reconstructed profile. Considering the 15‐mm circular field on‐axis dose as reference, the relative output factor for each field size can be defined as the ratio of the corresponding on‐axis dose to the reference dose.

**Figure 9:**
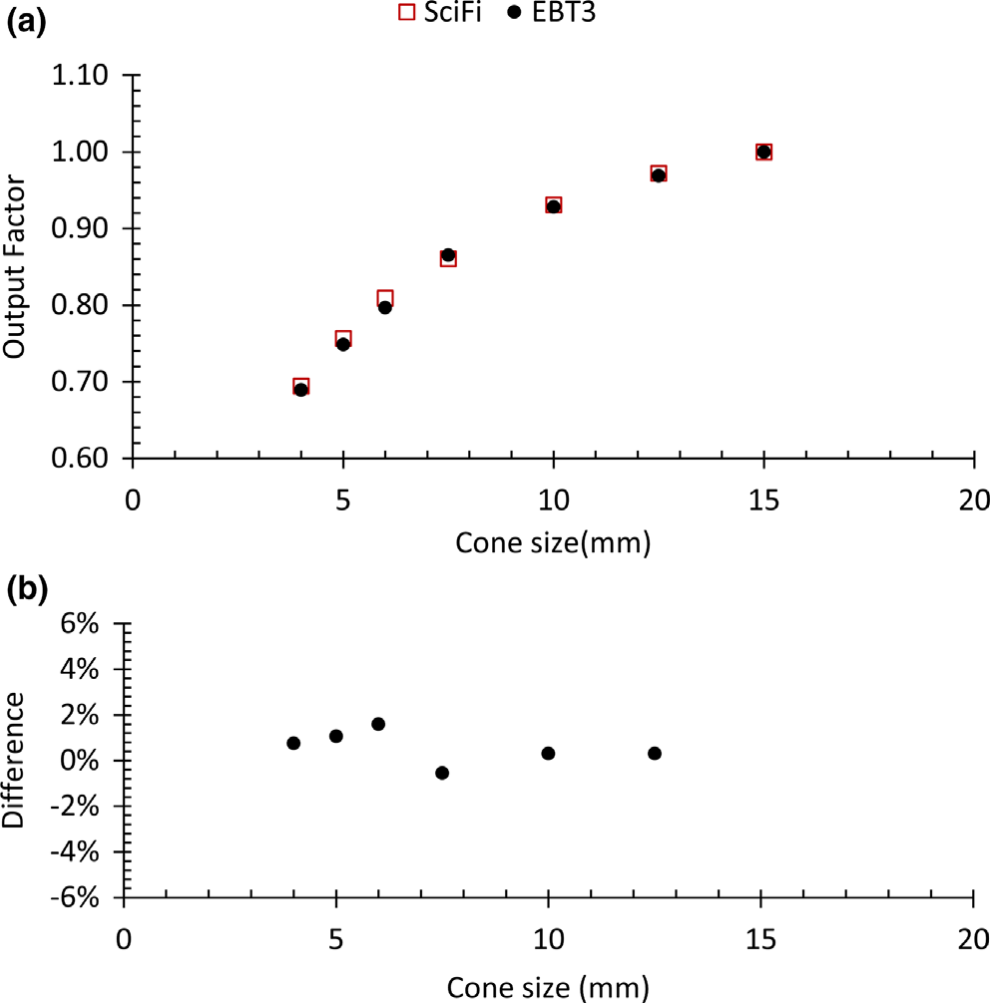
(a) Relative output factor for different cone sizes normalized to the 15‐mm cone size and (b) percentage local deviations of SciFi measurements as compared with Institute for radiological protection and nuclear safety data. [Color figure can be viewed at http://wileyonlinelibrary.com]

Figure 9(a) plots the relative output factor for different field sizes, obtained from the SciFi detector and from IRSN data acquired independently with EBT3 films and TLD according to their well‐established protocol.^14^, ^25^

Local deviations of SciFi measurements vs IRSN data are also plotted in percentage in Fig. 9(b). The differences remain within ±1.6% for all cone sizes.

## 4 Discussion

### 4.1 SciFi detector for real‐time cone‐based small‐field Quality Assurance

The obtained results show that the SciFi detector can be used to measure the beam profiles and output factors for stereotactic cone collimators, with enough sensitivity and resolution for real‐time monitoring of small fields down to 4 mm size.

The SciFi detector has some advantages over diode detectors which require corrections for the non‐water equivalence and/or volume‐averaging effects.^12^, ^24^

In comparison with the field profile method using a single cylindrical scintillating fiber for field scanning,^13^ the reconstructed profile using image acquisition from the SciFi detector allows real‐time field monitoring.

Our proposed measuring approach is compatible with MRI‐LINAC systems with potentially better performances than diodes which are not recommended for Output Factor measurements for such systems.^24^

The SciFi detector is also more adapted than radiochromic films for daily QA procedures of cone‐based stereotactic radiosurgery since films require careful handling and time‐consuming off‐line processing.^12^, ^14^

### 4.2 SciFi detector data processing for dose profile reconstruction

One specificity of the proposed technology is that the signal at the detector output is a projection of the field along the waveguide direction. For high‐resolution 2D dosimeter using long scintillating fibers, Goulet et al. have proposed the rotation of the detector and a tomographic reconstruction.^20^ In the case of cone‐based fields, our proposed reconstruction method does not require any rotation of the detector for dose profile and OF QA. This method simply uses some *a priori* knowledge of the dose distribution with the following assumptions: symmetry of revolution of the dose distribution, highest dose on the beam axis with decrease of the dose with distance from this axis.

The proposed method processes a single dose projection and thus allows a reduction of the size of the system of linear equations as compared with other algebraic reconstruction techniques. The computation time is significantly reduced, and the noise robustness of the reconstruction is improved.

Beam profile parameters (FWHM and penumbra width 20%–80%) as well as Output Factor based on reconstructed dose profiles give coherent results with the corresponding data evaluated with radiochromic films and TLD. The observed differences mean that these parameters can be accurately estimated with the proposed approach. On the other hand, it is possible to enhance signal to noise ratio by optimizing the readout chain at the SciFi detector output. This may allow to maintain full spatial resolution for images (instead of using 5 × 5 binned images) and to achieve higher spatial resolution on the beam parameters.

## 5 Conclusions

For online determination of beam parameters and OF for small static fields defined by stereotactic cones, we propose a new measuring approach with implementation based on the SciFi detector (initially developed for LHCb experiment at CERN). It includes a novel dose profile reconstruction method to process images from a single‐orientation detector in the field. The system has been tested in clinical conditions for small fields defined by using 4.5‐, 6‐, 7.5‐, 10‐, 12.5‐, and 15‐mm cone collimators. FWHM and penumbra have been determined from the reconstructed beam profile, with differences within ±0.32 mm as compared with EBT3 radiochromic film measurements. By optimizing the SciFi readout chain to enhance the signal to noise ratio, the spatial accuracy for determining beam profile parameters can further be improved.

For real‐time determination of the field output factor, the measured result from our implemented system are in good agreement with data independently determined by IRSN based on combined measurements with radiochromic films and TLD 1 × 1×1 mm^3^ micro‐cubes dosimeters. The differences are within ±1.6% for all cone sizes.

The proposed system fulfills the recommendation of IAEA‐AAPM TRS‐483 code of practice: To determine field output factors for small fields, the output correction factors of the detectors should be close to unity. We have employed the SciFi detector with no correction factor. It is also noted that the proposed technology and approach is compatible with MRI‐LINAC.

## Conflict of Interest

The authors have no relevant conflict of interest to disclose.

## References

[1] Duggan DM , Coffey CW . Small photon field dosimetry for stereotactic radiosurgery. Med Dosim. 1998;23:153–159. 9783268 10.1016/s0958-3947(98)00013-2

[2] Andreo P . The physics of small megavoltage photon beam dosimetry. Radiother Oncol. 2018;126:205–213. 29191457 10.1016/j.radonc.2017.11.001

[3] Palmans H , Andreo P , Huq MS , Seuntjens J , Christaki KE , Meghzifene A . Dosimetry of small static fields used in external photon beam radiotherapy: summary of TRS‐483, the IAEA–AAPM international code of practice for reference and relative dose determination. Med Phys. 2018;45:e1123–e1145. 30247757 10.1002/mp.13208

[4] Borzov E , Nevelsky A , Itzhak RB . Dosimetric characterization of Elekta stereotactic cones. J Appl Clin Med Phys. 2017;19:194–203. 29266744 10.1002/acm2.12242 PMC5768017

[5] Solberg TD , Balter JM , Benedict SH , et al. Quality and safety considerations in stereotactic radiosurgery and stereotactic body radiation therapy: executive summary. Pract Radiat Oncol. 2012;2:2–9. 25740120 10.1016/j.prro.2011.06.014 PMC3808746

[6] Klein DM , Tailor RC , Archambault L , Wang L , Therriault‐Proulx F , Beddar AS . Measuring output factors of small fields formed by collimator jaws and multileaf collimator using plastic scintillation detectors. Med Phys. 2010;37:5541–5549. 21089789 10.1118/1.3488981 PMC2962666

[7] Garnier N , Amblard R , Villeneuve R , et al. Detectors assessment for stereotactic radiosurgery with cones. J Appl Clin Med Phys. 2018;19:88–98. 30216702 10.1002/acm2.12449 PMC6236831

[8] Gershkevitsh E , Centre M , Huq MS . A novel method for the determination of field output factors and output correction factors for small static fields for six diodes and a microdiamond detector in megavoltage photon beams. Med Phys. 2019;46:944–963. 30521073 10.1002/mp.13318 PMC7379629

[9] Che K , Bulski W . A multi‐centre analytical study of small field output factor calculations in radiotherapy. Phys Imag Radiat Oncol. 2018;6:1–4 10.1016/j.phro.2018.03.001 PMC7807585 33458380

[10] Czarnecki D , Zink K . Monte Carlo calculated correction factors for diodes and ion chambers in small photon fields. Phys Med Biol. 2013;58:2431–2444. 23514734 10.1088/0031-9155/58/8/2431

[11] Benmakhlouf H , Sempau J , Andreo P . Output correction factors for nine small field detectors in 6 MV radiation therapy photon beams: a PENELOPE monte carlo study. Med Phys. 2014;41:1–12. 24694131 10.1118/1.4868695

[12] Gonzalez‐Lopez A , Lago‐Martin J‐D , Vera‐Sanchez J‐A . Small fields measurements with radiochromic films. J Med Phys. 2015;40:61. 26170551 10.4103/0971-6203.158667 PMC4478646

[13] Morin J , Béliveau‐Nadeau D , Chung E , et al. A comparative study of small field total scatter factors and dose profiles using plastic scintillation detectors and other stereotactic dosimeters: the case of the CyberKnife. Med Phys. 2013;40:1–11. 10.1118/1.4772190 23298089

[14] Institute for Radiological Protection and Nuclear Safety (IRSN) ‐ Rapport N° PSE‐SANTE/SDOS/2018‐00035 ‐ Dosimétrie Des Mini‐Faisceaux Mise à Jour Du Protocole Dosimétrique de Détermination Des FOC Dans Les Mini‐ FaisceauxUtilisés En Radiothérapie; 2018; http://logi103.xiti.com/go.click?xts=:410711%26s2=3%26p=PSE-SAN-SDOS-2018-00035-Mini-faisceaux%26clic=T%26type=click%26url=http://www.irsn.fr/FR/expertise/rapports_expertise/Documents/radioprotection/IRSN_PSE-SAN-SDOS-2018-00035_Mini-faisceaux-FOC.pdf

[15] Hopchev P . SciFi – A large scintillating fibre tracker for LHCb. The Fifth Annual Conference on Large Hadron Collider Physics Shanghai Jiao Tong. University, Shanghai, China May; 2017.

[16] Joram C , Uwer U , Leverington BD , et al. LHCb Scintillating Fibre Tracker Engineering Design Review Report: Fibres. Geneva: Mats and Modules; 2015 https://cds.cern.ch/record/2004811

[17] Bassinet C , Huet C , Derreumaux S , et al. Small fields output factors measurements and correction factors determination for several detectors for a CyberKnife ® and linear accelerators equipped with microMLC and circular cones. Med Phys. 2013;40:071725. 23822429 10.1118/1.4811139

[18] Archambault L , Briere TM , Beddar S . Transient noise characterization and filtration in CCD cameras exposed to stray radiation from a medical linear accelerator. Med Phys. 2008;35:4342–4351. 18975680 10.1118/1.2975147 PMC2736755

[19] Jin X , Hirakawa K . Analysis and processing of pixel binning for color image sensor. EURASIP J Adv Signal Process. 2012;2012:1–15.

[20] Goulet M , Archambault L , Beaulieu L , Gingras L . High resolution 2D dose measurement device based on a few long scintillating fibers and tomographic reconstructiona. Med Phys. 2012;39:4840–4849. 22894410 10.1118/1.4736526

[21] van Aarle W , Palenstijn WJ , Cant J , et al. Fast and flexible X‐ray tomography using the ASTRA toolbox. Opt Express. 2016;24:25129. 27828452 10.1364/OE.24.025129

[22] Sorzano COS , Vargas J , Otón J , et al. A Survey of the use of iterative reconstruction algorithms in electron microscopy. Biomed Res Int. 2017;2017:1–17. 10.1155/2017/6482567 PMC5623807 29312997

[23] Gaudette RJ , Brooks DH , Dimarzio CA , et al. A comparison study of linear reconstruction techniques for diffuse optical tomographic imaging of absorption coefficient. Phys Med Biol. 2000;45:1051–1070. 10795991 10.1088/0031-9155/45/4/318

[24] Petasecca M , Al Shukaili K , Pereveratylo V , Lerch M , Jackson M , Rosenfeld A . Characterization of ELEKTA SRS cone collimator using high spatial resolution monolithic silicon detector array. J Appl Clin Med Phys. 2018;19:114–124. 10.1002/acm2.12345 PMC6036391 29790261

[25] Bassinet C , Huet C . Rapport IRSN PSE‐SANTE/SDOS N° 2017‐00014: Détermination Des FOC Sur Une Installation de Radiothérapie Délivrant Des Mini‐Faisceaux ‐ Accélérateur Truebeam Équipé de CollimateursConiques Du. Service de Radiothérapie Des Hospices Civils de Lyon; 2017.

